# Effects of High-Pressure, Hydrothermal, and Enzyme-Assisted Treatment on the Taste and Flavor Profile of Water-Soluble Ginger (*Zingiber officinale*) Extract

**DOI:** 10.3390/foods11040508

**Published:** 2022-02-10

**Authors:** Dong-Geon Nam, Mina Kim, Jeong-Sook Choe, Ae-jin Choi

**Affiliations:** Division of Functional Food & Nutrition, Department of Agrofood Resources, National Institute of Agricultural Science, Rural Development Administration, Wanju-gun 55365, Korea; realfood@korea.kr (D.-G.N.); lucidminakim@gmail.com (M.K.); swany@korea.kr (J.-S.C.)

**Keywords:** *Zingiber officinale*, enzyme-assisted water-soluble extraction, electronic nose, SBSE-TD/GC-MS/MS, taste, volatiles

## Abstract

Ginger, a plant widely consumed worldwide, is used as a spice or to enhance the flavor of foods. In this study, the taste characteristics (gingerol, shogaol, and amino acid) of extracts treated with various solubilizing methods were objectively compared. In addition, an E-nose confirmed the flavor pattern combined with principal component analysis (PCA) between each extract gas chromatogram-tandem mass spectrometry was performed to compare and analyze volatile compounds between extraction methods. As a result, high-pressure enzyme-assisted extraction (HPE) and hydrothermal enzyme-assisted extraction (HWE) treatment effectively improved the extraction yield of ginger and the contents of gingerol and shogaol and removed the bitter taste. In addition, radar charts of both E-nose and PCA provided the distribution of flavor substances in HPE and HWE products of ginger. After enzyme-assisted treatment, a strong fruity and piquant flavor was noted. In conclusion, it is suggested that ginger extract of enzyme-assisted treatment has increased flavor compounds and can be an excellent food material.

## 1. Introduction

In modern food, the form and quality are affected by the environment, disease, or culture of a society. In addition, natural raw material cultivation technology and processing technology have influence [1,2]. Consumers prefer products that do not contain chemicals or additives or that are produced using environmentally friendly processing technology, which satisfies the natural environment and health at the same time [3]. The association between processing technology and flavor has a significant impact on product quality [4]. Flavor control during processing requires a high level of skill due to the complexity of the reaction and the formulation or process conditions [5]. Extraction is a method of obtaining certain constituents of an organism from compounds with various matrices [6,7]. In order to increase the extraction efficiency from plants, it is necessary to identify the characteristic structure and properties of plants, and to homogenize it accordingly [8,9]. Most solubilized bioactive compounds are in the form of glycosides; these forms are easy for bio-digestion and absorption and affect action and active effects [10].

Because of these advantages, bioactive compounds have been extracted by various methods from general households to industries and are still being used in various ways. Existing extraction techniques include maceration, distillation, decoction, etc. and have disadvantages in the use of a large amount of organic solvent, low yield, high cost, and long extraction time [11,12,13]. In order to compensate for these shortcomings, aqueous extraction [14], supercritical [15], high-pressure [16], ultrasound [17], microwave [18], and enzyme-assisted [19] extraction have been performed and are attractive for solubilizing phytochemical compounds. Due to the high cost of extraction equipment such as high-pressure, supercritical, ultrasonic, and microwave instruments, this process is difficult to perform on small-scale farms or small and medium-sized factories. On the other hand, aqueous enzyme-assisted treatment is relatively easy to apply and environmentally safe for extracting bioactive components [20,21]. The structure of the ginger cell wall is similar to that of other root crops, in which starches such as cellulose and structural proteins are intricately intertwined [22]. In addition, enzyme-assisted treatment is easy to use in combination with other methods as one way to increase the storage properties of ginger. In this study, a combination of enzyme-assisted extraction was tested as a relatively new process for solubilizing an increased active component and flavor profile from ginger.

Ginger is a commonly known medicinal root crop and has a distinctive flavor and taste due to its composition of volatile compounds and other components. Volatile compounds are the most important factor in evaluating the flavor of food. Typical flavor components of ginger include α-zingiberene, β-sesquiphellandrene, and β-bisabolene, [23,24] which are monoterpenoid and sesquiterpene (β-sesquiphellandrene, β-bisabolene, etc.) volatile hydrocarbons mainly present in the free form. In addition, non-volatile phenols (oleoresin) are composed of gingerols, shogaols, and zingerone (α-zingiberene) [25]. Gas chromatography-tandem-mass-spectrometry is useful for detecting and identifying volatile substances, but it is not useful for analyzing the properties or pattern information of the identified substances. Instrumental flavor analysis can utilize material states and components through smell rather than subjective analysis using artificial sensory evaluation. Considering that the electronic nose (E-nose) can obtain the overall volatile component information of a sample, analysis in combination with other instruments can be useful for the study of flavor [26]. Electronic noses have been widely used for flavor detection, quality monitoring, and freshness and spoilage evaluation [27,28].

In this study, the taste characteristics (gingerol, shogaol, and amino acid) of extracts treated with various solubilizing methods were objectively compared. In addition, E-nose confirmed the flavor pattern combined with PCA between each extract, and SBSE-TD/GC-MS/MS was performed to compare and analyze volatile compounds between extraction methods. 

## 2. Materials and Methods

### 2.1. Materials

#### 2.1.1. Enzymes and Chemicals

Enzymes: pectinase (pectinex Ultra SP-L, ≥3800 PGNU/mL) and α-amylase (termamyl 2X, 240 KNU/g) were purchased from Novo Nordisk (Bagsvaerd, Denmark).

Chemicals: ethanol, methanol, acetonitrile, sodium phosphate, sodium tetraborate, γ-aminobutyric acid (GABA), and taurine were purchased from Sigma-Aldrich Co. (St. Louis, MO, USA). 6-gingerol (6G), 8-gingerol (8G), 10-gingerol (10G), 6-shogaol (6S), 8-shogaol (8S), and 10-shogaol (10S) were purchased from Chromadex (Laguna Hills, CA, USA). Aspartic acid (Asp), glutamic acid (Glu), serine (Ser), histidine (His), glycine (Gly), threonine (Thr), alanine (Ala), arginine (Arg), tyrosine (Tyr), valine (Val), methionine (Met), phenylalanine (Phe), isoleucine (Iso), leucine (Leu), lysine (Lys), proline (Pro), glutamine (Gln), asparagine (Asn), tryptophan (Try), ornitnine (Orn), citrulline (Cit), borate buffer, o-phthalaldehyde reagent, and 9-fluorenylmethylchloroformate reagent were purchased from Agilent (Santa Clara, CA, USA).

#### 2.1.2. Plant Materials and Sample Preparation

Fresh ginger (*Zingiber officinale* Rosc.) was harvested from a farm in Bong-dong, Jeollabuk-do Province, South Korea. The ginger was peeled, washed, and hot-air dried. The dried pieces were cut into slices, pulverized, and stored at –20 °C until use in the experiment.

### 2.2. Extraction

The hydrothermal and high-pressure enzyme-assisted method has been described in our previous study [29]. The various extraction methods of ginger used in the present experiment are shown in Table 1.

#### 2.2.1. Squeezed Raw Ginger Juice and Tea

In addition to the dried form, fresh ginger was ground in a mixer and squeezed (GJ). Ginger tea was prepared with the hot-air-dried ginger powder (HDP, 5 g) at 80 °C for 30 min in 95 mL distilled water (GT).

#### 2.2.2. Hydrothermal Extraction

Hydrothermal enzyme-assisted extraction (HWE) was studied using two enzymes, pectinase (breaks down pectin in the cell wall through hydrolysis) and α-amylase (hydrolysate of starch). The HDP (5 g) was extracted in a transparent pouch with distilled water (95 mL) and pectinase (1% *w*/*w*, substrate contrast). The enzymatic reaction was performed in a shaking water bath (WiseBath, MaXturdy, Daihan Scientific, Wonju, Korea) at 50 °C for 2 h. After the treated ginger mixture was transferred to a beaker, α-amylase (1% *w*/*w*, substrate contrast) was added. The enzymatic reaction was performed in a water bath at 93 °C for 1 h. Finally, the reaction was terminated by boiling the ginger mixture for 5 min and centrifuging (Labogene, Gyro1580MGR, Gyrogen Co., Ltd., Daejeon, Korea) at 2863× *g* for 10 min. Hydrothermal extraction (HW) was performed without enzyme for comparison to the HWE method. The supernatants were filtered and stored at 4 °C for timely use. In this study, enzyme co-treatment was performed without adjusting the pH. In other studies, pH adjustment did not significantly affect the composition of the extract [30].

#### 2.2.3. High-Pressure Extraction

High-pressure extraction (HP) and high-pressure enzyme-assisted extraction (HPE) were performed using the same procedure as for the HWE and HW described in Section 2.2.2. However, for cell wall hydrolysis, an ultra-high-pressure liquefaction instrument (Chemresys Co., Anyang, Korea) was used at 50 °C, 100 MPa, and 2 h instead of a water bath.

### 2.3. Extraction Yield and Sugar Content Measurement

The extraction yields of raw ginger and ginger extraction were calculated on a dry weight basis by dividing the quantity of the obtained product into the initial weights of raw ginger and HDP. Sugar content (Bx°) was measured using a refractometer (ATAGO, PAL-1, Tokyo, Japan). All analyses were conducted in triplicate.

### 2.4. Analysis of Taste Compounds

#### 2.4.1. Gingerols and Shogaols Analysis

The standards and ginger extracts were analyzed on a Shimadzu UPLC system (Nexera X2, Shimadzu, Kyoto, Japan) using the specifications of [30]: injection volume, 2 μL; flow rate, 0.3 mL/min; retention time, 30 min; wavelength, 280 nm; and eluents, 0.1% acetic acid in water (A) and 0.1% acetic acid in acetonitrile (B). The gradient elution had the following profile: 0–0.5 min, 90% A; 0.5–2.5 min, 60% A; 2.5–4.5 min, 45% A; 4.5–6.0 min, 40% A; 6.0–11.5 min, 35% A; 11.5–13.0 min, 30% A; 13.0–14.5 min, 25% A; 14.5–16.0 min, 20% A; 16.0–17.5 min, 15% A; 17.5–25.0 min, 10% A; 25.0–30.0 min, 90% A. In the UPLC analysis, a Kinetex XB.C18 column (1.7 μm, 150 × 2.1 mm, Phenomenex, Torrnace, CA, USA) was used and the column temperature was maintained at 30 °C. The concentrations in each sample were calculated by comparing their response with the corresponding standard curve. The calibration curve for this method has the six calibration standards solutions (6G, 8G, 10G, 6S, 8S, and 10S) each covering the range of 10–1000 μg/mL (calculation by the equations (R2 coefficients): 6G; y = 942.59x + 16,123 (0.9993), 8G; y = 781.68x + 9155 (0.9994), 10G; y = 752.09x + 10,667 (0.9994), 6S; y = 1103.3x + 13,039 (0.9994), 8S; y = 944.76x + 16,414 (0.9994), 10S; y = 558.24x + 6124 (0.9995)).

#### 2.4.2. Free Amino Acid (AA) Analysis

Amino acid analysis was referred to the method of [31]. The standards and ginger extracts also were analyzed on a Thermo HPLC system (Thermo Scientific, Karlsruhe, Germany) with operating parameters of injection volume, 0.5 μL; flow rate, 1.5 mL/min; retention time, 35 min; wavelength, 338 nm; and eluents, 40 mM sodium phosphate, pH 7 (A) and ice-cold lysis/extraction buffer (methanol/acetonitrile/water, 4.5/4.5/1 *v*/*v*) (B). The gradient elution had the following profile: 0–3.0 min, 95% A; 3.0–24.0 min, 45% A; 24.0–31.0 min, 10% A; 31.0–35.0 min, 95% A. In the HPLC analysis, a Inno C18 column (5 μm, 150 × 4.6 mm, Youngjin Biochrom, Seongnam, Korea) was used and the column temperature was maintained at 40 °C. A reference amino acid spectrum was obtained by titrating a mixture of amino acids of known concentrations.

### 2.5. Analysis of Volatile Compounds

#### 2.5.1. Electronic Nose Analysis

The volatile compounds in gingers extracted through the different methods were measured using an HERACLES II electronic nose (E-nose) (Alpha MOS, Toulouse, France). The analysis condition was referred to as spirit beverage [32]. Briefly, 20 mL of ginger extract sample was sealed in a glass vial and heated to 40 °C for 20 min at an agitation speed of 500 rpm. Helium was used as the carrier gas, with a flow rate of 1 mL/min. A total volume of 5000 μL was injected into the system at 200 °C. The volatile compounds were absorbed by an embedded volatile concentrator named Tenax TA at 20 °C for 30 s with a split mode of 10 mL/min, and thermal desorption was performed at 240 °C for 30 s. An MXT-5 column was employed for sample separation in parallel mode. The programmed temperature was 50 °C for 2 s, increased to 80 °C at 1 °C/s, and ramped to 250 °C at 3 °C/s for 21 s. The temperature of both flame ionization detectors were set to 260 °C. All the samples were performed in 5 repeats.

#### 2.5.2. SBSE-TD/GC-MS/MS Analysis

GC–MS/MS was performed on a 7890 B gas chromatograph coupled to a 7000 triple quadrupole mass spectrometer (Agilent Technologies, Palo Alto, CA, USA) system equipped with an Agilent Multimode injector (Agilent Technologies, Palo Alto, CA, USA). The instrument control, data acquisition and analysis were performed with Agilent Mass-Hunter software (Agilent Technologies, Palo Alto, CA, USA). A stir bar coated with a 0.1 mm layer of non-polar polydimethylsiloxane (PDMS, 10 mm × 0.1 mm film thickness) was purchased from Gerstel (Mullheim an der Ruhr, Germany). We injected 20 mL of ginger extract for each condition into a 20 mL vial containing a PDMS-coated stir bar. The vial was sealed and stirred at 800 rpm for 5 h at room temperature. The stir bar was removed from the solution and transferred to a thermal desorption liner (Gerstel, Mullheim an der Ruhr, Germany), mounted on a TDU (Gerstel, Mullheim an der Ruhr, Germany) and analyzed. The PDMS adsorption equilibrium period was determined by a pre-test (pre-test results not shown) [33]. The analysis condition was applied to peach juice [34] and bay berry juice [35]. Chromatographic separations were carried out using a DB–5MS column (60 m × 0.32 mm ID, 0.25 μm) supplied by Agilent Technologies (Palo Alto, CA, USA). Helium and nitrogen were used as quenching gas with a constant flow rate of 1.5 mL/min and collision gas with a constant flow rate of 1.0 mL/min, respectively. Purge flow of the cushion was set as 1.5 mL/min. The injector temperature was 230 °C. Oven temperature program was held at 40 °C for 20 min, ramped with 10 °C/min to 130 °C and was held for 5 min, then ramped with 10 °C/min to 200 °C and was held for 5 min, and ramped with 10 °C/min to 300 °C and was finally held for 5 min. The temperature of the electronic ionization source and the transmission line were 230 °C, and the mass spectrum was obtained by electronic impact at 70 eV. Each volatile compound was represented by an area unit.

### 2.6. Statistical Analysis

All measurements (contents of yield, sugar, gingerols, shogaols, and amino acids, area of volatile compounds) were performed in triplicate except for the E-nose analysis (5 replicates). One-way ANOVA and Duncan’s test were applied to analyze the difference between the means of volatile compounds, and the difference between groups was analyzed using the independent-sample t-test. SPSS version 18.0 (Statistical Package for Social Sciences, Chicago, IL, USA) was used to process chemical composition, amino acid, and E-nose analysis data. SIMCA^®^ version 17 software (Sartorious, Goettingen, Germany) was used to construct PCA, VIP score, and multivariate analysis data to evaluate the ability of GC-MS/MS to discriminate between samples.

## 3. Results and Discussion

### 3.1. Extraction Yield and Sugar Contents

The extraction yields of the ginger and sugar contents are shown in Table 1. The extraction yields obtained for HPE (67.60%) and HWE (55.40%) were significantly higher than those of GJ (1.11%) and GT (16.26%) (*p* < 0.001) and higher than those obtained after extraction from freeze-dried ginger powder by reflux extraction (hydrothermal, 100 °C, 2 h, and 15.35%) [29]. The complex extraction method increased the extraction yield. The sugar content of the ginger extract was significantly higher in HWE (2.57 Bx°) and HPE (2.53 Bx°) (*p* < 0.001). In general, the enzyme- and non-enzyme extract showed many difference in sugar content. These differences are affected by the sample type, temperature, time, substrate and enzyme concentration, etc [29,30]. Enzymatic hydrolysis increased the extraction yield and sugar content.

### 3.2. Taste Compounds Properties

#### 3.2.1. Spiciness: Gingerol and Shogaol

The components that impart “spiciness” to ginger are gingerols and shogaols, secondary metabolites of the Zingiberaceae family [36]. Shogaol is both spicy and sweet in flavor [37]. In general, spiciness is subjectively evaluated by several factors (type and temperature of food, race, psychological state, living environment, etc.) [38]. Depending on the amounts of extracted gingerol and shogaol compounds, the degree of spiciness can be inferred objectively. Extraction yield indicates that treatment at high-pressure can be more effective than hydrothermal treatment (Table 2). In HDP, the level of 6G increased in HWE and HPE by 5.5 times and 5.0 times, respectively, compared to GT. In addition, 6G, 8G, 10G, 6S, 8S, and 10S showed significant differences between the ginger extract samples (*p* < 0.001). This combination of enzymes has shown an effect on gingerol and shogaol extraction from Korean ginger [30]. The gingerol and shogaol compounds demonstrated stability in aqueous solution [39]. The effect of high-pressure treatment was demonstrated in beet extract [40] and citrus peel extract [41]. Each gingerol and shogaol compound was affected by the extraction yield of ginger. In particular, the higher was the shogaol content, the stronger was the spicy taste. As a result, gingerol and shogaol were affected by the extraction yield, and there was a slight difference between the high-pressure treatment and the hydrothermal treatment. For applications in industry, high-pressure methods are expensive, whereas hydrothermal preparation is relatively cheap. HWE treatment is very effective in increasing the yield and functional components at the same time. With ginger, considering the production cost, it is judged that hydrothermal treatment is suitable for enzyme-assisted treatment. 

#### 3.2.2. Bitterness, Umami, Salty, Sourness and Sweetness: Free Amino Acids

Protein is broken down or formed by internal and external enzymes to produce various amino acids that determine taste. AA and peptides are important determinants of taste [42]. Among the known AAs, Pro, Val, Leu, Tyr, Phe, and Arg are bitter; Glu and Asp are umami; and Thr, Ala, and Gly are sweet [43]. The AAs corresponding to bitter, umami, and sweet were effectively removed in the enzyme-assisted treatment group (HWE and HPE) (Table 3). In addition, the yield with HWE was lower than that of GJ, and some components in HPE were lower than those in HPE. After identifying the amounts of non-essential AAs (Gln, Asn, Glu, Arg, Ala, Pro, Asp, Ser, Gly, and Try), higher amounts were detected in HW and HP extracts compared to GT extract. As for Glu, which is often expressed as umami taste, HW and HP increased 1.5 to 1.6 times that of GT, respectively, and significantly increased from 2.8 to 3.0 times that of GJ. Val and Leu were smaller in essential AA than in Nigerian ginger, and non-essential AAs were slightly different as Glu, Arg, Ala, and Asp [44]. For other AAs, GT was significantly higher in Orn (43.19 mg/100 g), Cit (33.98 mg/100 g), and GABA (56.97 mg/100 g) (*p* < 0.001). The pectinase (cell wall degrading enzyme) used in this study was mainly used for clarification of fruit and vegetable juice. The clarification effect of plant extracts or juices can be explained as a result of high molecular compounds in plants forming oxidized proteins and partially removing neutral sugars (galacturonic acid monomers) [45,46]. Previous studies have demonstrated that pectinase is superior as an enzyme that breaks down the cell wall of ginger [29]. In addition, plant proteins hydrolyzed by enzymes or hydrothermal treatment are associated with AAs, peptides, minerals, and volatile compounds [47]. The content of AA affects the flavor according to the treatment method, and the decrease in the content of AA related to taste was shown to result from enzyme treatment [5]. A method for selectively removing AA corresponding to taste has not yet been developed. These results suggest that the enzyme-assisted treatment effectively removes the bitter AA of ginger and neutralizes other tastes, offering a positive effect on industrial applications.

### 3.3. Volatile Compounds Properties

#### 3.3.1. Radar Charts and PCA by E-nose

**Figure 1:**
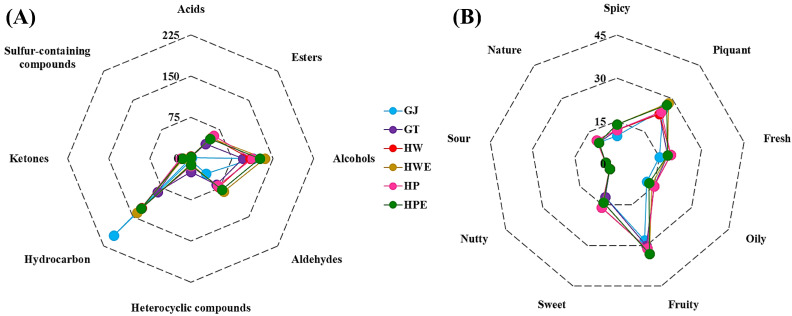

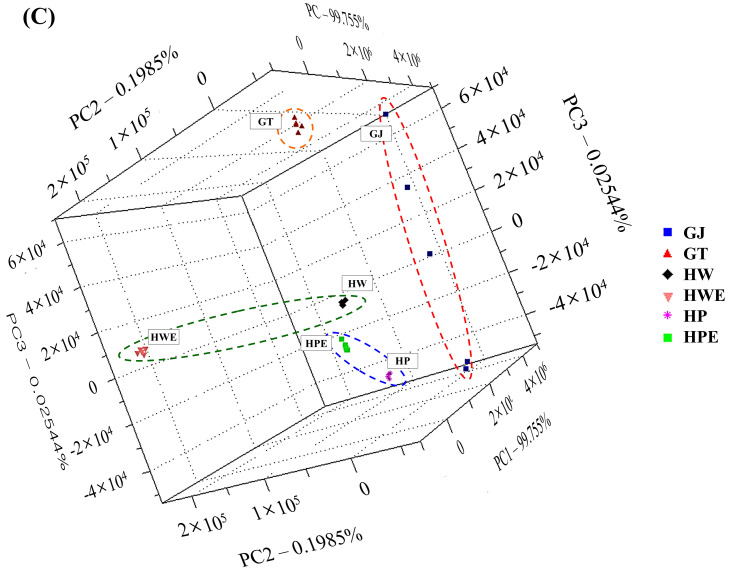
E-nose results of ginger extracts for 6 types of pre-treatment processes. Radar chart of area units by E-nose (**A**). Radar chart of sensory descriptors by E-nose (**B**). 3D model of PCA based on the E-nose data set (**C**). GJ, squeezed raw ginger; GT, ginger tea; HW, hydrothermal extraction; HWE, hydrothermal enzyme-assisted extraction; HP, high-pressure extraction; HPE, high-pressure enzyme-assisted extraction.

The flavor of plants is mainly generated by volatile components, and the flavor components can be altered through extraction or processing. The main flavor component of extracted and processed ginger has a great influence on the quality of the product and its taste. To identify odor substances contributing to ginger, the E-nose was applied to analyze the volatile compounds and sensory properties of the samples. This analysis was also performed on soy bean paste [51] and jujube [52] to further elucidate the ability of volatile compounds to discriminate samples. A total of 47 volatile components was investigated in raw ginger and five ginger extracts with various extraction methods were applied, 45 in GJ, 45 in GT, 47 in HW, 46 in HWE, 47 in HP, and 47 in HPE. In total, 47 volatile components were detected, including 3 acids, 6 alcohols, 8 aldehydes, 11 esters, 2 aromatic heterocyclic compounds, 10 terpene hydrocarbons, 5 ketones, and 2 aromatic sulfur-containing compounds. The number of volatile compounds in each sample, determining the sensory properties, are shown in Figure 1, and details of the volatile composition are given in Supplementary Material. As shown in Figure 1A and Table S1, the HWE sample had the highest area of volatile compounds, whereas the GT had the lowest. This result indicates the stronger flavor provided by HWE compared to the other five samples. Figure 1B and Table S2 shows the analyzed sensory characteristics as spicy, piquant, fresh, oily, fruity, sweet, nutty, sour, and nature, which correspond to food. The calculated flavor characteristics were classified into 12 spicy, 27 piquant, 19 fresh, 15 oily, 31 fruity, 16 sweet, 3 nutty, 4 sour, and 11 natures. The number of flavor words expressing each ginger sample was 119 words for GJ, 127 words for GT, 134 words for HW, 138 words for HWE, 135 words for HP, and 136 words for HPE. Compared to GJ, each extract showed an increase in spicy, fresh, fruity, and sweet. On the other hand, piquancy increased in HWE- and HPE-treated with enzymes. Variables affecting each sample were explored using PCA, one of the multivariate analysis methods, and the separated result pattern is shown in Figure 1C and Table S3. PCA accounted for 99.76% (PC1), 0.20% (PC2), and 0.03% (PC3) of the overall variance. Squeezed raw ginger juice and five different extracts were well isolated from the PCA. GT and HP samples showed certain similarities in flavor properties, and HW, HWE and HPE samples showed similar flavor profiles. Therefore, the E-nose is an effective tool to discriminate the flavor patterns of ginger extract.

#### 3.3.2. Comprehensive Analysis of SBSE-TD/GC-MS/MS Volatile Compounds

**Figure 2:**
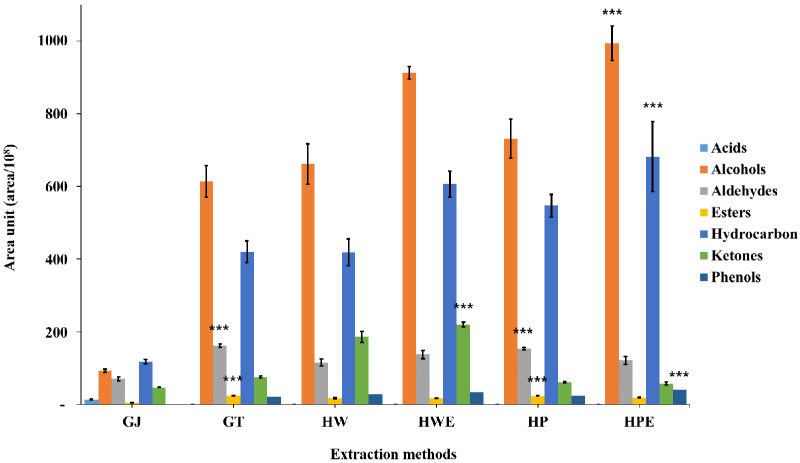
Volatile compounds identified by SBSE-TD/GC-MS/MS of ginger extracts for 6 types of pre-treatment processes. Significance was compared with one-way ANOVA between the extraction method and the area of compound classification; GJ, squeezed raw ginger; GT, ginger tea; HW, hydrothermal extraction; HWE, hydrothermal enzyme-assisted extraction; HP, high-pressure extraction; HPE, high-pressure enzyme-assisted extraction; ***, *p* < 0.001.

Ginger samples from different extraction methods were analyzed using the SBSE-TD/GC-MS/MS technique to evaluate the volatile compounds in the extract. A total of 54 types of volatile components was investigated in 6 ginger samples, and volatile components of 41 types of GJ, 30 types of GT, 33 types of HW, 32 types of HWE, 35 types of HP, and 34 types of HPE were detected. Volatile compounds were mainly found as 2 acids, 18 alcohols, 6 aldehydes, 1 ester, 21 hydrocarbons, 4 ketones, and 2 phenols. As shown in Figure 2 and Table S4, the HPE sample had the highest area of volatile compounds, while the lowest was observed in GJ. HDP enriches the aromatic compounds. In ginger, the area of the flavor component increased according to the extraction method. As a result of comparison by extraction method and each volatile compound classification, HPE showed the significantly highest levels in alcohols, hydrocarbons, and phenols; HWE was highest in ketones; and GT and HP were highest in aldehydes and esters (*p* < 0.001). The extraction method that produced the strongest flavor was HPE, and the enzyme-assisted HPE and HWE showed relatively rich flavors compared to the other sample groups.

As for acid compounds, two were detected only in GJ, n-hexadecanoic acid (palmitic acid) and octadecanoic acid (stearic acid), which are saturated fatty acids commonly found in vegetable oils. The components accounted for 12 (area%, 3.48%) of the total component ratio.

Alcohol was observed in compounds 11, 11, 11, 13, 13, and 15, respectively, in the GJ, GT, HW, HWE, HP, and HPE samples. Alcohol was the most abundant group in HPE, accounting for 1040 (53.84%) of the total peak area, and GJ was the lowest at 95 (26.77%). The components of terpenes (eucalyptol, linalool, borneol, terpineol, ocimenol, cubebanol, nerolidol, and acorenol, etc.) appearing in spice plants such as flowers and plants were detected. Specifically, a kind of insect pheromone verbenol was detected in the extract groups except GJ. Monoterpenes and sesquiterpenes are volatile terpenoids that have been reported in various plant types [53,54,55].

Aldehyde compounds were observed in the GJ, GT, HW, HWE, HP, and HPE samples as 4, 4, 4, 3, 5, and 4 compounds, respectively. Aldehydes were the most abundant group in GJ, accounting for 73 (20.53%) of the total peak area, and HPE had the lowest at 125 (6.49%). The more complicated was the extraction method, the lower was the ratio of aldehyde compounds. Among the detected aldehyde compounds, components such as citral and neral, which are flavor components specific to ginger, showed a high ratio of GJ.

As for the ester compound, one compound was observed in all sample groups. The detected ester compound is bornyl acetate, which is a perfume compound corresponding to an odor. At the same time, this compound was reduced in HWE and HPE treated with enzymes.

Hydrocarbon compounds were observed in different amounts in the samples, with 18, 10, 12, 10, 12 and 10 compounds detected in the GJ, GT, HW, HWE, HP and HPE samples, respectively. The relative area ratios of hydrocarbon compounds detected in each sample ranged from 122 (34.37%) to 460 (29.47%). HPE was significantly higher among the treatment groups (*p* < 0.001). In GJ, the components of monoterpenes (β-phellandrene and β-terpinene) were increased, but these components were not detected in four extracts (HW, HWE, HP, and HPE) except GT. The α-terpinene level was smaller than that of GJ in area ratio. Hydrocarbon compounds such as α-funebrene were additionally found in ginger extracted with hot water at 120 °C or higher [56].

Ketone compounds were observed in different amounts in the samples, with 4, 2, 3, 3, 2, and 2 compounds detected in the GJ, GT, HW, HWE, HP, and HPE samples, respectively. The relative area ratio of ketone compounds detected in each sample was 47 (13.18%), 203 (13.03%), and 217 (12.12%) for GJ, HW, and HWE, respectively, and 78 (5.57%), 59 (3.53%), and 59 (3.05%) for GT, HP, and HPE. The level of Zingerone, a ketone component showing the sweet flavor of ginger, was found to be high in the hydrothermal treatment (HW 203 and HWE 217).

Only one phenolic compound was detected in GJ and two compounds each in GT, HW, HWE, HP, and HPE. The relative area ratio of phenolic compounds (6-gingerone and 6-shogaol) detected in each sample was highest in HPE at 39 (2.04%) and lowest in GJ at 1 (0.27%). Among the phenolic compounds, shogaol was the spiciest flavor of ginger and was detected in the range of 0.97% to 1.54% in the sample group excluding GJ. These results are the effects of hot-air drying on ginger. Heating to high temperature converts dehydrated-hydrated gingerol to shogaol [41].

Overall, the HDP extract was rich in alcohol compounds, and GJ had more aldehyde compounds in the extract group. Volatile compounds (zingiberenol, 6-gingerone, and 6-shogaol) corresponding to spicy flavor were further increased by enzyme extraction. Acid compounds were detected only in GJ, and phenolic compounds were high in HPE. These results can be attributed to the processing and extraction conditions of ginger. In HDP, alcohol and aldehyde compounds additionally detected components with a specific insect pheromone odor. These flavor components are inferred as rancid flavor (sour flavor) in Figure 1. Various conditions for solubilization of ginger were major in eliciting aromatic compounds. In the case of mushrooms, it was reported that a unique flavor is formed by the interaction of several volatile compounds with chemical components [57]. The flavor profile of extracts from dry powders has rarely been studied. In conclusion, the increase in flavor compounds of the ginger extract by enzyme treatment suggests that it can be used as an excellent food industry application material.

#### 3.3.3. Comparison of Total Volatile Compounds by Extraction Method

**Figure 3:**
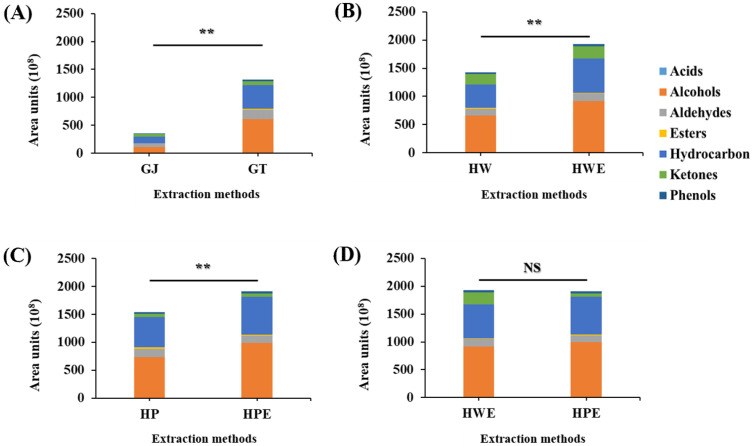
Comparison of total volatile compounds by extraction method. Significance was compared by independent-sample t-tests between the extraction methods. Area units of volatile compounds; between GJ and GT (**A**); between HW and HWE (**B**); between HP and HPE (**C**); between HWE and HPE (**D**); GJ, squeezed raw ginger; GT, ginger tea; HW, hydrothermal extraction; HWE, hydrothermal enzyme-assisted extraction; HP, high-pressure extraction; HPE, high-pressure enzyme-assisted extraction; **, *p* < 0.01; NS, not significant.

The results compared by the various extraction methods are shown in Figure 3. The volatile compounds by sample type are shown in Figure 3A, and the flavor of the samples infused with HDP in hydrothermal leaching was significantly increased. Thermal drying by air can seriously affect the plant epidermis and cells, causing structural deformation [58]. Gingerol in ginger is converted to 6S as water is removed by physical factors, and other components are converted into the same molar amounts of gingerol and hexanal by rearrangement [59,60]. In ginger, ingredients with a spicy flavor, such as shogaol, can affect consumer preference. Regardless of hydrothermal and high-pressure, the enzyme-assisted samples had a significant increase in flavor compounds (Figure 3B,C), and there was no effect of high-pressure treatment (Figure 3D). In a study of Australian ginger treated with supercritical carbon dioxide, heat drying reduced alcohol compounds (geraniol and citronellol) while significantly increasing the concentrations of monoterpene and sesquiterpene hydrocarbon compounds [61,62]. The enzyme-assisted treatment effectively enhanced flavor compounds.

#### 3.3.4. Different Extraction Methods Affected Ginger Extract Flavor Compounds

**Figure 4:**
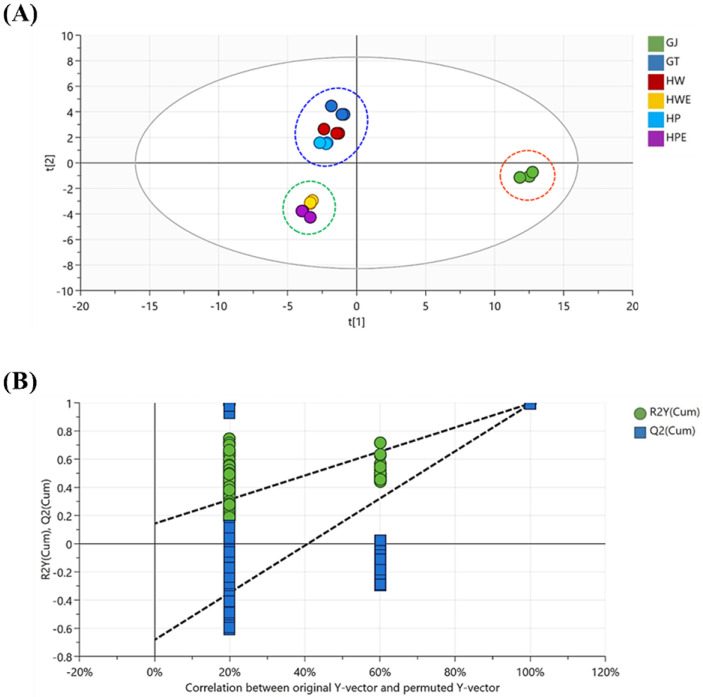
Volatile compounds in ginger extract for 6 types of pre-treatment processes. GJ, squeezed raw ginger; GT, ginger tea; HW, hydrothermal extraction; HWE, hydrothermal enzyme-assisted extraction; HP, high-pressure extraction; HPE, high-pressure enzyme-assisted extraction. The PCA score plot based on the GC-MS/MS data set (**A**). The PLS-DA results of permutation tests (**B**).

**Figure 5:**
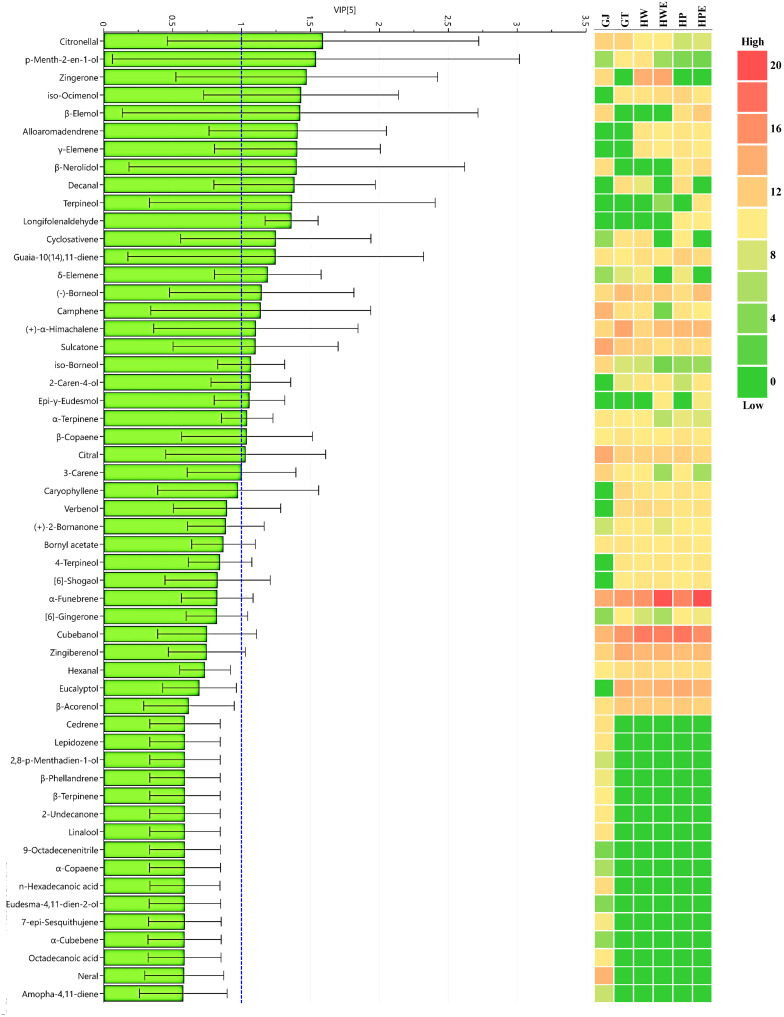
Volatile characteristics of ginger extract for 6 types of pre-treatment processes by PLS-DA; GJ, squeezed raw ginger; GT, ginger tea; HW, hydrothermal extraction; HWE, hydrothermal enzyme-assisted extraction; HP, high-pressure extraction; HPE, high-pressure enzyme-assisted extraction; VIP > 1; *p* < 0.05.

The extraction method can have a significant effect on the flavor compounds in ginger. This analysis was also performed on limequat fruit [63] and garlic [64] to further elucidate the ability of volatile compounds to discriminate samples. The score plot shows a greater distance between GJ and GT, HW, and HP and between GT, HW, HP, and HWE, HPE, the greater is the change in volatile compounds with extraction method. Further analysis of volatile ginger compounds changes by various extraction methods with PLS-DA. Figure 4B is the result of a permutation test with a permutation number of 500. The permuted green R2 on the left of the graph showed a value of 0.143, and the blue Q2 had a value of –0.683, lower than the original point on the right, indicating the validity of the PLS-DA model. In conclusion, the comparison between ginger extraction methods showed that the PLS-DA model is suitable and has high discriminating power and predictability. Figure 5 and Table S4 shows the screened major volatile constituents of ginger based on VIP values (VIP > 1) and *p*-values (*p* < 0.05) by ANOVA. A total of 24 types of volatile compounds corresponding to a VIP score of 1 or more were found, and the citronellal compound had the highest score and the highest influence. The analyzed results further showed the non-negligible effect of the extraction method on the volatile constituents of ginger.

## 4. Conclusions

In this study, the taste characteristics (gingerol, shogaol, and amino acid) of extracts treated with various solubilizing methods were objectively compared. In addition, the E-nose confirmed the flavor pattern combined with PCA between each extract, and SBSE-TD/GC-MS/MS was performed to compare and analyze volatile compounds between extraction methods. HPE and HWE significantly improved the extraction yield of ginger and the contents of gingerol and shogaol, and enzyme treatment effectively removed the bitter taste. The flavors of six types of ginger extracts varied by method, and a total of 54 volatile compounds was identified. After HWE treatment, a strong fruity and piquant flavor was reported. Enzymatic hydrolysis of ginger contributed to the aromatic substances of citronellal, zingerone, and citral. In addition, the spicy flavor was increased as the phenolic components of 6-gingerone and 6-shogaol increased. HWE treatment effectively improved the quality of ginger to a level suitable for additive or industrial applications.

## Figures and Tables

**Table 1:** Extraction yield and sugar content of ginger extract after 6 types of pre-treatment processes.

Pre-Treatment Method	Symbol	Yield (%)	Bx°
Squeezed juice/raw	GJ	1.11 ± 0.01 ^f^	1.10 ± 0.00 ^e^
Hot-water/leached/powder	GT	16.26 ± 0.18 ^e^	1.83 ± 0.06 ^b^
Hydrothermal/powder	HW	22.66 ± 0.23 ^d^	1.20 ±0.00 ^d^
Hydrothermal/enzyme/powder	HWE	55.40 ± 1.18 ^b^	2.57 ± 0.06 ^a^
High-pressure/powder	HP	24.46 ± 0.19 ^c^	1.40 ± 0.00 ^c^
High-pressure/enzyme/powder	HPE	67.60 ± 0.48 ^a^	2.53 ± 0.06 ^a^
	F-value	6575.07 ***	767.53 ***

Data represent mean value ± standard deviation. Vertically, lowercase letters represent significant content differences between extracts (*p* < 0.05). Bx°, sugar contents; ***, *p* < 0.001.

**Table 2:** Gingerol and shogaol contents of ginger extracts after 6 types of pre-treatment processes.

	GJ	GT	HW	HWE	HP	HPE	F-Value
6G (mg/g)	0.81 ± 0.00 ^f^	1.27 ± 0.02 ^e^	2.28 ± 0.03 ^c^	7.03 ± 0.01 ^a^	2.18 ± 0.03 ^d^	6.30 ± 0.01 ^b^	50,470.25 ***
8G (mg/g)	0.17 ± 0.00 ^f^	0.22 ± 0.00 ^e^	0.50 ± 0.00 ^c^	1.67 ± 0.00 ^a^	0.47 ± 0.00 ^d^	1.53 ± 0.00 ^b^	79,353.60 ***
10G (mg/g)	0.48 ± 0.00 ^f^	0.73 ± 0.01 ^e^	1.37 ± 0.01 ^c^	4.08 ± 0.01 ^a^	1.31 ± 0.01 ^d^	3.60 ± 0.03 ^b^	52,907.18 ***
6S (mg/g)	0.29 ± 0.00 ^f^	0.44 ± 0.00 ^e^	0.86 ± 0.00 ^c^	2.52 ± 0.01 ^b^	0.83 ± 0.00 ^d^	2.62 ± 0.01 ^a^	116,407.04 ***
8S (mg/g)	0.00 ± 0.00 ^d^	0.00 ± 0.00 ^d^	0.04 ± 0.00 ^c^	0.39 ± 0.00 ^b^	0.04 ± 0.00 ^c^	0.41 ± 0.00 ^a^	10,460.00 ***
10S (mg/g)	0.15 ± 0.01 ^c^	0.22 ± 0.02 ^c^	0.48 ± 0.03 ^b^	1.44 ± 0.09 ^a^	0.46 ± 0.03 ^b^	1.44 ± 0.01 ^a^	574.91 ***

Data represent mean value ± standard deviation. Horizontally, lowercase letters represent significant content differences between extracts (*p* < 0.05). GJ, squeezed raw ginger; GT, ginger tea; HW, hydrothermal extraction; HWE, hydrothermal enzyme-assisted extraction; HP, high-pressure extraction; HPE, high-pressure enzyme-assisted extraction; 6G, 6-gingerol; 8G, 8-gingerol; 10G, 10-gingerol; 6S, 6-shogaol; 8S, 8-shogaol; 10S, 10-shogaol; ***, *p* < 0.001.

**Table 3:** Taste, intensity, and amino acid content of ginger extract for the 6 types of pre-treatment processes.

		Intensity	Taste	GJ	GT	HW	HWE	HP	HPE	F-Value
Essential amino acid (mg/100 g)	Val	Slight	Bitter	14.38 ± 0.51 ^c^	86.39 ± 3.88 ^a^	81.31 ± 1.46 ^b^	6.74 ± 1.58 ^d^	76.92 ± 4.59 ^b^	2.14 ± 0.27 ^d^	725.61 ***
	Leu	Extreme	Bitter	7.33 ± 0.27 ^d^	41.85 ± 1.61 ^c^	50.03 ± 0.98 ^a^	2.45 ± 0.22 ^e^	47.02 ± 2.41 ^b^	1.56 ± 0.02 ^e^	1049.34 ***
	Iso	Moderate	Bitter	6.31 ± 0.23 ^d^	38.31 ± 1.56 ^c^	44.04 ± 1.07 ^a^	1.18 ± 0.21 ^e^	41.15 ± 2.32 ^b^	0.70 ± 0.02 ^e^	893.61 ***
	Lys	Slight	Bitter, Salty	3.37 ± 0.19 ^e^	53.76 ± 1.04 ^a^	47.89 ± 0.49 ^b^	5.87 ± 0.29 ^d^	44.04 ± 2.24 ^c^	2.52 ± 0.12 ^e^	1689.51 ***
	Thr	Slight	Sweet	11.32 ± 0.35 ^d^	50.25 ± 2.22 ^a^	46.91 ± 0.90 ^b^	0.99 ± 0.02 ^e^	43.68 ± 2.78 ^c^	1.14 ± 0.05 ^e^	739.05 ***
	Phe	Extreme	Bitter	4.57 ± 0.16 ^c^	28.48 ± 1.07 ^b^	31.16 ± 0.61 ^a^	1.88 ± 0.17 ^d^	29.51 ± 1.59 ^b^	2.19 ± 0.02 ^d^	957.72 ***
	Met	Extreme	Bitter	5.32 ± 0.21 ^d^	7.18 ± 0.08 ^b^	7.31 ± 0.13 ^ab^	6.43 ± 0.22 ^c^	7.71 ± 0.46 ^a^	0.40 ± 0.01 ^e^	411.70 ***
	His	Extreme	Bitter	11.28 ± 0.34 ^c^	12.39 ± 0.36 ^c^	19.95 ± 0.43 ^a^	0.17 ± 0.02 ^d^	18.04 ± 1.49 ^b^	0.36 ± 0.04 ^d^	487.65 ***
	Tyr	Extreme	Bitter	7.82 ± 0.28 ^c^	71.61 ± 3.73 ^b^	86.40 ± 1.45 ^a^	1.53 ± 0.14 ^d^	81.94 ± 4.97 ^a^	2.32 ± 0.02 ^d^	777.03 ***
Non-essential amino acid (mg/100 g)	Gln	Slight	Sweet, Salty, Bitter	0.22 ± 0.01	ND	2.19 ± 0.10	ND	0.77 ± 0.17	ND	NS
	Asn	-	Tasteless	ND	ND	120.94 ± 2.60	ND	108.90 ± 3.79	ND	NS
	Glu	Extreme	Umami, Sweet	81.21 ± 2.82 ^c^	154.42 ± 4.00 ^b^	239.90 ± 5.43 ^a^	7.62 ± 0.30 ^d^	230.30 ± 16.38 ^a^	7.63 ± 0.06 ^d^	609.76 ***
	Arg	Moderate	Bitter	0.13 ± 0.02 ^e^	3.49 ± 1.94 ^cd^	77.63 ± 1.30 ^a^	4.83 ± 0.15 ^c^	69.70 ± 2.63 ^b^	0.94 ± 0.02 ^de^	1988.54 ***
	Ala	Moderate	Umami, Sweet	45.59 ± 1.57 ^b^	181.41 ± 7.18 ^a^	189.35 ± 3.50 ^a^	3.76 ± 0.12 ^c^	180.19 ± 11.48 ^a^	3.85 ± 0.09 ^c^	772.78 ***
	Pro	Moderate	Bitter, Sweet	7.29 ± 0.48 ^c^	2.02 ± 0.58 ^d^	70.56 ± 1.64 ^a^	0.66 ± 0.03 ^de^	64.03 ± 0.57 ^b^	0.56 ± 0.01 ^e^	5646.38 ***
	Asp	Extreme	Umami	93.46 ± 3.16 ^c^	24.27 ± 2.34 ^d^	245.80 ± 4.19 ^a^	3.66 ± 0.19 ^e^	230.38 ± 12.73 ^b^	3.13 ± 0.03 ^e^	1156.88 ***
	Ser	Moderate	Sweet	49.24 ± 1.67 ^c^	6.82 ± 0.34 ^d^	119.63 ± 3.04 ^a^	1.19 ± 0.09 ^e^	102.99 ± 3.25 ^b^	1.38 ± 0.05 ^e^	2251.65 ***
	Gly	Moderate	Sweet	8.00 ± 0.32 ^c^	77.48 ± 3.55 ^a^	57.46 ± 1.59 ^b^	0.62 ± 0.05 ^d^	54.13 ± 3.06 ^b^	1.32 ± 0.03 ^d^	836.63 ***
	Try	Slight	Bitter	8.46 ± 0.13 ^b^	10.70 ± 4.61 ^b^	12.11 ± 1.09 ^ab^	14.98 ± 0.07 ^a^	10.80 ± 1.90 ^b^	2.38 ± 0.13 ^d^	12.53 ***
Other amino acid (mg/100 g)	Orn	Slight	Sour	5.18 ± 0.11 ^de^	43.19 ± 1.03 ^a^	29.13 ± 1.75 ^b^	6.58 ± 0.15 ^d^	26.15 ± 1.94 ^c^	3.25 ± 0.14 ^e^	605.70 ***
	Cit	Extreme	Sour	26.91 ± 0.92 ^b^	33.98 ± 1.71 ^a^	11.08 ± 0.43 ^d^	18.87 ± 0.16 ^c^	10.68 ± 0.73 ^d^	1.61 ± 0.10 ^e^	560.48 ***
	GABA	-	Tasteless	6.47 ± 0.21 ^c^	56.97 ± 1.85 ^a^	56.42 ± 1.17 ^ab^	1.78 ± 0.09 ^d^	53.53 ± 3.34 ^b^	1.18 ± 0.02 ^d^	935.86 ***

Data represent mean value ± standard deviation. Horizontally, lowercase letters represent significant content differences between extracts (*p* < 0.05). For the taste and intensity of each amino acid, refer to [48,49,50]; GJ, squeezed raw ginger; GT, ginger tea; HW, hydrothermal extraction; HWE, hydrothermal enzyme-assisted extraction; HP, high-pressure extraction; HPE, high-pressure enzyme-assisted extraction; Val, valine; Leu, leucine; Iso, isoleucine; Lys, lysine; Thr, threonine; Phe, phenylalanine; Met, methionine; His, histidine; Tyr, tyrosine; Gln, glutamine; Asn, asparagine; Glu, glutamic acid; Arg, arginine; Ala, alanine; Pro, proline; Asp, aspartic acid; Ser, serine; Gly, glycine; Try, tryptophan; Orn, ornitnine; Cit, citrulline; GABA, γ-aminobutyric acid; ***, *p* < 0.001; NS, not significant; ND, not detected; -, no data.

## Data Availability

Not applicable.

## Supplementary Materials

The following are available online at https://www.mdpi.com/article/10.3390/foods11040508/s1, Table S1: Area units by E-nose; Table S2: Sensory descriptors words count by E-nose; Table S3: Volatile compounds identified in samples by E-nose; Table S4: Volatile compounds identified in samples by GC-MS/MS.
