# Supplementary material for: Effects of High-Pressure, Hydrothermal, and Enzyme-Assisted Treatment on the Taste and Flavor Profile of Water-Soluble Ginger (Zingiber officinale) Extract
Source: Foods. 2022 Feb 10;11(4):508. doi: 10.3390/foods11040508 (PMC8871348; doi:10.3390/foods11040508)
Supplement: Supplementary file 1 [file foods-11-00508-s001.zip › foods-1581309-supplementary.pdf]

foods

Supplementary Materials

Table S1. Area units by E-nose.

Area/10³	GJ	GT	HW	HWE	HP	HPE
Acids	0	3	4	3	4	4
Esters	2	38	54	53	59	50
Alcohols	114	96	108	135	113	126
Aldehydes	39	67	73	85	69	80
Heterocyclic compounds	3	24	12	10	14	12
Hydrocarbon	199	86	127	139	129	129
Ketones	1	16	16	13	19	16
Sulfur-containing compounds	0	0	1	1	1	1

GJ, squeezed raw ginger; GT, ginger tea; HW, hydrothermal extraction; HWE, hydrothermal enzyme-assisted extraction; HP, high-pressure extraction; HPE, high-pressure enzyme-assisted extraction.

Table S2. Sensory descriptors words count by E-nose.

Sensory words	GJ	GT	HW	HWE	HP	HPE
Spicy	10	12	12	14	12	14
Piquant	25	23	23	28	24	27
Fresh	15	18	19	18	19	18
Oily	12	15	15	14	15	13
Fruity	28	30	31	33	31	33
Sweet	12	12	16	14	16	14
Nutty	3	3	3	3	3	3
Sour	4	4	4	4	4	4
Nature	10	10	11	10	11	10
Total	119	127	134	138	135	136

GJ, squeezed raw ginger; GT, ginger tea; HW, hydrothermal extraction; HWE, hydrothermal enzyme-assisted extraction; HP, high-pressure extraction; HPE, high-pressure enzyme-assisted extraction.

Table S3. Volatile compounds identified in samples by E-nose.

No.	Classification	Compounds	RT	RI	Sensory description	GJ		GT		HW		HWE		HP		HPE	
						Mean	±SD	Mean	±SD	Mean	±SD	Mean	±SD	Mean	±SD	Mean	±SD
4	Acid and esters	Pentane	16.60	491	Oily	218.05	3.79	6852.37	41.76	10632.76	183.89	11409.64	63.29	13881.06	225.30	10046.62	248.94
8	Acid and esters	Formic acid	21.47	605	Acidic, Pungent, Vinegar	52.98	1.30	1397.93	4.78	2004.20	37.60	1515.90	27.24	2205.32	19.41	2056.87	21.45
9	Acid and esters	Acetic acid	22.69	614	Acidic, Pungent, Sour, Vinegar	48.89	0.34	1303.51	27.64	1705.58	60.62	1741.51	110.01	1312.64	5.57	1421.40	6.15
10	Acid and esters	Diisopropyl ether	23.12	620	-	33.41	0.57	1531.21	20.81	1132.02	25.83	3721.21	16.04	3798.20	38.56	2862.47	42.60
11	Acid and esters	Methylcyclopentane	24.00	630	Acidic, Pungent, Sour, Vinegar	44.00	0.53	557.35	4.92	805.35	98.77	878.87	13.72	976.75	17.65	867.85	19.51
15	Acid and esters	Heptane	30.19	701	Fruity, Sweet	17.59	0.86	1814.25	33.24	2540.77	239.74	2269.90	59.33	2949.45	29.21	2678.31	32.28
16	Acid and esters	Methylcyclohexane	33.92	732	Fruity, Sweet	ND	ND	ND	ND	159.48	29.96	119.48	1.38	106.52	6.23	145.24	6.89
17	Acid and esters	2-methylpropanoic acid	35.18	742	Acidic, Butter, Cheese, Dairy, Pungent, Rancid, Sour	10.15	0.05	121.35	4.25	268.78	44.82	185.88	7.07	180.42	8.49	270.90	9.39
21	Acid and esters	Octane	42.16	803	Fruity, Sweet	145.67	2.06	12704.29	161.73	16527.08	9.18	14052.36	216.82	18353.11	241.90	16894.84	267.28
24	Acid and esters	4-Ethylheptane	46.90	847	-	92.66	1.53	1534.01	15.87	1856.55	23.70	1451.71	6.54	1586.82	34.53	1508.63	38.16
25	Acid and esters	Ethyl isovalerate	48.46	863	Apple, Fruity, Pineapple, Sweet	24.21	1.37	70.87	6.00	56.51	5.18	56.70	2.16	58.65	1.52	ND	ND
30	Acid and esters	Nonane	53.93	920	Oily	43.65	0.50	158.34	11.04	117.13	39.24	124.12	7.73	114.98	7.09	114.22	7.84
42	Acid and esters	Ethyl cyclohexanecarboxylate	69.70	1134	Cheese, Fruity	612.83	38.87	1938.05	56.41	1469.38	222.46	1505.05	167.32	1578.70	107.72	744.34	119.03
46	Acid and esters	Butyl octanoate	84.30	1414	Butter, Floral, Fruity, Green, Oily	954.91	25.96	15623.27	553.28	18610.61	187.25	17440.22	131.61	15743.14	1286.77	14671.61	1421.75
18	Alcohols	2-penten-1-ol	38.29	769	Green, Mushroom	27.71	1.58	604.15	6.69	762.68	24.49	117.97	2.51	581.75	38.28	672.71	42.30
23	Alcohols	2,3-dimethyl-1-Pentanol	44.68	826	Leek, Onion, Sulfurous	6.69	0.10	151.57	12.16	180.21	2.33	132.95	4.41	242.11	11.52	247.63	12.73
33	Alcohols	1-Heptanol	57.83	968	Aromatic, Fresh, Nutty	106318.61	573.63	38729.88	1321.15	25444.20	866.91	41985.67	272.40	26825.10	350.42	32755.54	387.18
38	Alcohols	1-Octanol	65.46	1073	Floral, Fresh, Green, Orange, Sulfurous	971.01	10.03	1432.53	53.78	823.67	23.88	617.73	15.84	855.47	69.40	722.76	76.69
41	Alcohols	2,6-dimethyl-Phenol	68.64	1120	Rooty, Sweet	313.40	1.70	619.37	32.19	464.74	17.24	412.27	21.07	660.14	42.80	558.62	47.29
47	Alcohols	3-Tetradecanol	93.69	1603	Dairy	7717.56	236.05	97493.50	2630.84	82454.07	3484.65	92800.97	3412.27	94748.18	5770.57	92300.83	6375.91
2	Aldehydes	Acetaldehyde	14.45	438	Fresh, Fruity, Pleasant, Pungent	282.99	23.29	1851.45	54.32	3248.89	126.55	6448.44	199.95	4285.93	261.83	4317.02	289.30
3	Aldehydes	Propanal	15.57	464	Cocoa, Earthy, Pungent	2075.46	48.81	5509.35	335.96	12559.25	500.01	18874.13	282.72	8601.35	400.87	21618.53	442.92
5	Aldehydes	2-methylpropanal	17.80	515	Floral, Fresh, Fruity, Pungent, Spicy	45.69	1.08	798.17	15.15	668.20	20.52	1014.04	11.23	505.93	4.67	1115.04	5.16
12	Aldehydes	2-Butenal	26.08	653	Floral, Green, Pungent	17.13	0.91	10143.80	41.52	12465.65	49.12	10110.80	101.06	16013.34	216.39	11568.90	239.09
34	Aldehydes	2,4-Heptadienal	60.19	1007		33325.28	1369.38	34750.53	1117.50	25846.62	437.27	21946.37	810.06	29942.60	357.50	24236.38	395.00
40	Aldehydes	n-Nonanal	67.41	1107	Citrus, Floral, Fresh, Fruity, Green, Melon, Orange, Orange peel, S	2776.64	17.76	11212.36	81.05	7627.92	90.87	7054.01	73.65	9267.90	72.31	7922.04	79.90
44	Aldehydes	Decanal	74.08	1211	Citrus, Floral, Green, Lemon, Orange, Orange peel, Sweet	241.20	8.07	3355.21	67.21	2383.73	28.00	2318.56	35.89	2845.33	48.99	2591.98	54.13
45	Aldehydes	Cinnamaldehyde	77.20	1268	Apple, Cinnamon, Pungent, Spicy, Sweet	1426.59	177.63	8982.81	388.16	7134.81	135.35	6612.27	131.48	7651.24	584.33	6823.38	645.63
36	Heterocyclic compounds	Acetylpyrazine	62.55	1030	Corn, Nutty	1715.64	12.27	1533.09	91.72	1064.68	29.46	862.01	20.21	1215.59	29.82	940.86	32.94
43	Heterocyclic compounds	2-Methoxy-3-(1-methylpropyl)pyrazir	72.05	1179	Earthy, Green, Pepper	1512.23	128.71	22383.46	1016.92	10893.55	186.27	9179.31	123.64	12420.51	261.98	10755.69	289.47
1	Hydrocarbon	Ethyl chloride	13.91	424	Pungent	16.66	0.60	253.13	18.07	225.25	6.22	ND	ND	199.73	24.78	ND	ND
6	Hydrocarbon	Acetonitrile	19.43	553	Aromatic, Sweet	61.86	1.85	7724.51	28.14	9314.23	48.89	7761.88	49.79	8302.23	48.89	7355.43	54.01
19	Hydrocarbon	1-ethyl-3-methyl-Cyclopentane	39.04	775	Pungent	119.58	10.03	1084.34	17.68	1122.96	66.88	760.31	19.44	1373.88	21.02	1289.89	23.22
26	Hydrocarbon	Ethylbenzene	49.60	874	Aromatic, Floral, Sweet	7.12	0.32	ND	ND	58.82	4.16	63.07	0.00	61.63	1.62	ND	ND
27	Hydrocarbon	1,4-dimethylbenzene	50.34	880	Aromatic	11.79	0.73	217.97	6.75	198.30	32.77	147.63	8.90	171.91	4.42	161.16	4.88
31	Hydrocarbon	Butylcyclopentane	55.46	938	-	2202.54	27.30	872.62	54.97	746.78	42.52	593.96	16.97	1008.49	32.90	610.63	36.35
32	Hydrocarbon	4-Methylnonane	56.35	949	-	41749.30	499.08	13268.01	520.24	12372.00	51.22	9213.89	311.69	15418.00	220.06	9478.75	243.14
35	Hydrocarbon	(1-Methylpropyl)benzene	61.74	1018	-	9929.15	27.96	4518.11	91.08	3748.25	62.52	3037.51	87.07	4341.51	29.73	2945.58	32.85

37	Hydrocarbon	γ-Terpinene	63.81	1049	Citrus, Fruity, Herbaceous, Lemon, Oily, Sweet	145936.82	396.63	91321.44	4785.31	99406.07	2061.64	117031.41	1320.84	97849.64	2085.04	106411.14	2303.77
39	Hydrocarbon	Terpinolene	66.37	1085	Citrus, Fresh, Fruity, Herbaceous, Sweet	ND	ND	972.50	51.51	625.13	37.67	516.92	49.49	854.42	66.40	802.05	73.36
7	Ketones	Butanone	20.92	592	Butter, Cheese, Fruity, Pleasant, Pungent, Sweet	82.68	0.53	1053.30	23.48	1794.37	52.58	992.20	72.50	1683.02	28.49	1941.71	31.48
13	Ketones	1-Hydroxy-2-propanone	26.98	664	Pungent, Sweet	31.45	0.99	10368.57	64.28	10388.34	78.23	8320.65	87.36	13157.46	136.04	9819.19	150.31
14	Ketones	2-Pentanone	29.15	689	Fruity, Sweet	9.67	0.02	166.62	2.52	282.68	2.05	59.79	2.81	111.48	1.40	300.57	1.54
28	Ketones	2-Heptanone	51.67	893	Banana, Cheese, Coconut, Fruity, Musty, Nutty, Spicy, Sweet	219.37	14.89	1564.84	33.78	1331.24	19.94	1150.33	15.37	1521.65	7.28	1270.63	8.05
29	Ketones	Crotonolactone	52.55	901	Butter	205.55	3.95	3255.26	66.28	2486.95	54.73	2187.46	21.54	2819.62	29.84	2533.98	32.97
20	Sulfur-containing compoun	2,3-Butanediol	41.13	792	Creamy, Fruity, Onion	0.00	0.00	337.42	8.29	626.09	35.62	566.31	16.20	609.68	20.87	522.81	23.06
22	Sulfur-containing compoun	3-methyl-2-butene-1-thiol	43.96	819	Leek, Onion, Sulfurous	4.79	0.28	129.74	4.54	157.54	15.57	148.89	1.08	150.29	9.68	147.26	10.70

GJ, squeezed raw ginger; GT, ginger tea; HW, hydrothermal extraction; HWE, hydrothermal enzyme-assisted extraction; HP, high-pressure extraction; HPE, high-pressure enzyme-assisted extraction; RT, represents the retention time in the capillary GC column; RI, retention index using a MXT-5 column; Each value is expressed as mean ± SD (n = 3); ND, not detected; "-", not described.

Table S4. Volatile compounds identified in samples by GC-MS/MS.

No	Classification	Compound name	RT	RI	GJ		GT		HW		HWE		HP		HPE	
					Area	±SD	Area	±SD	Area	±SD	Area	±SD	Area	±SD	Area	±SD
50	Acids	n-Hexadecanoic acid	47.47	1968	903727459	41236194	ND	ND	ND	ND	ND	ND	ND	ND	ND	ND
52	Acids	Octadecanoic acid	50.68	2172	358465882	42077085	ND	ND	ND	ND	ND	ND	ND	ND	ND	ND
7	Alcohols	Eucalyptol	26.93	1032	ND	ND	11098401194	824896312	9700982897	882698838	15141261601	1403359982	12912353435	1087041906	14011450849	235483940
9	Alcohols	p-Menth-2-en-1-ol	28.61	1021	116254970	5965687	1274557898	79423158	2432961193	114482859	638380938	17277540	357675573	32675720	381345675	6188726
10	Alcohols	Linalool	28.69	1099	585748730	11044178	ND	ND	ND	ND	ND	ND	ND	ND	ND	ND
13	Alcohols	iso-Borneol	30.42	1157	1049014763	63872215	675141298	27437069	694170178	42706521	395576764	56578307	458790315	54883815	618692972	9691196
14	Alcohols	(-)-Borneol	30.52	1167	933153176	16826508	8115301653	262372488	5622655474	538863155	8664744494	405999162	3697694847	244821676	11286612499	522178745
15	Alcohols	4-Terpineol	30.53	1315	ND	ND	1897963632	67053061	1498215237	175788372	3064360291	81878547	1491263517	109401600	3041886813	137227651
16	Alcohols	iso-Ocimenol	30.86	1169	ND	ND	1810831744	484224753	2565259134	277933118	4856817541	173486496	5401513601	284482916	2421853851	200019599
17	Alcohols	Terpineol	30.98	1190	ND	ND	ND	ND	ND	ND	571541354	12750617	ND	ND	2770837610	571401182
19	Alcohols	Verbenol	31.89	1140	ND	ND	3456319885	165029095	4574286466	281266748	4911437521	76902214	2442040315	217375534	3806425325	170585380
21	Alcohols	2-Caren-4-ol	32.24	1181	ND	ND	744674094	62215603	1976928336	162013290	2587898697	115955309	703371639	54594914	2496160778	313106428
22	Alcohols	2,8-p-Menthadien-1-ol	32.41	1140	166355529	3048524	ND	ND	ND	ND	ND	ND	ND	ND	ND	ND
41	Alcohols	Cubebanol	39.44	1642	2411800404	333780383	14638392599	2534557508	20757945350	1754579076	26740718477	1675748723	23464472994	1157797326	23518778632	4018817004
42	Alcohols	β-Elemol	39.83	-	1033582047	19348185	ND	ND	ND	ND	ND	ND	3161763618	549729292	8621197854	1138205548
43	Alcohols	β-Nerolidol	39.96	1544	849556744	38203920	ND	ND	ND	ND	ND	ND	2321549642	385706354	5509273926	1218135030
44	Alcohols	Zingiberenol	40.88	-	1318466767	23899291	11539363120	417044248	9781772880	835378052	14529540566	1466306115	9379694654	957524327	12675145933	1137327329
45	Alcohols	Epi-γ-Eudesmol	40.93	1598	ND	ND	ND	ND	ND	ND	1225175261	72017956	ND	ND	1175387019	301849782
46	Alcohols	β-Acorenol	41.12	1649	668108314	12037686	6074477655	253468476	6555784714	457299528	7899617143	629882614	7292557827	1659763780	7077554797	118940879
48	Alcohols	Eudesma-4,11-dien-2-ol	42.49	1688	87830324	4476392	ND	ND	ND	ND	ND	ND	ND	ND	ND	ND
1	Aldehydes	Hexanal	9.53	800	296784524	5420164	3021462855	153357643	4281461849	328545520	4726720789	466880250	3266287905	150454355	4210923574	70703385
12	Aldehydes	Citronellal	29.88	1153	1249069351	178626474	4935705547	99395688	1056243013	103493808	1486459685	331090350	735627404	54619140	1000910174	16781509
18	Aldehydes	Decanal	30.99	1206	ND	ND	3093083175	64823668	804837530	30747327	ND	ND	3707102892	547293738	ND	ND
20	Aldehydes	Neral	32.18	1240	2532896786	393491149	ND	ND	ND	ND	ND	ND	ND	ND	ND	ND
23	Aldehydes	Citral	33.13	1276	2979545724	53652179	5,086,012,270	211267729	5359672806	463963213	7509010083	982508149	6406796681	671467437	5577536672	1141109233
49	Aldehydes	Longifolenaldehyde	42.65	1581	ND	ND	ND	ND	ND	ND	ND	ND	1202526810	39320948	1309131251	21882273
24	Esters	Bornyl acetate	33.48	1285	455534777	50876640	2337417633	76353583	1681446654	128472585	1712783762	44744723	2300672293	59333035	1854469038	114353719
2	Hydrocarbon	3-Carene	22.46	1011	1245650488	235902502	1167134362	84955625	1136830502	66988338	649587623	16737243	1240471358	129000810	678638592	49179904
3	Hydrocarbon	Camphene	23.60	952	2563790752	394025879	2933872937	104875382	2552189265	109522563	399471944	9244724	2628530600	552066041	1430218404	235915457
4	Hydrocarbon	β-Phellandrene	24.62	1031	202780484	3734721	ND	ND	ND	ND	ND	ND	ND	ND	ND	ND
5	Hydrocarbon	β-Terpinene	24.77	-	261875762	4870865	ND	ND	ND	ND	ND	ND	ND	ND	ND	ND
8	Hydrocarbon	α-Terpinene	28.33	1189	457753886	8247434	1067862668	43078235	1100189377	69372108	811615686	96805662	875138969	28684142	994775620	16713969
26	Hydrocarbon	δ-Elemene	34.93	1597	110691395	2046206	704890077	34136489	857415761	31808606	ND	ND	898546879	29407034	ND	ND
27	Hydrocarbon	α-Cubebene	35.27	1351	101461598	11683501	ND	ND	ND	ND	ND	ND	ND	ND	ND	ND
28	Hydrocarbon	Cyclosativene	35.94	-	103563059	3583170	2390834162	506393993	3121248161	118398087	ND	ND	1332882456	41615486	ND	ND
29	Hydrocarbon	α-Copaene	36.08	1376	131332037	4257850	ND	ND	ND	ND	ND	ND	ND	ND	ND	ND
30	Hydrocarbon	Guaia-10(14),11-diene	36.43	1461	643285107	11821466	1155224474	116578104	3368643310	240292966	2989010413	77019348	6387604720	209184384	5281751749	1080177759
31	Hydrocarbon	7-epi-Sesquithujene	36.66	1523	211583602	15379339	ND	ND	ND	ND	ND	ND	ND	ND	ND	ND
32	Hydrocarbon	β-Copaene	37.13	1376	252998416	11054319	2267776980	275535214	1237926802	118191807	1518479165	336010042	2738108730	82838514	2368414835	431823588
33	Hydrocarbon	γ-Elemene	37.34	1434	ND	ND	ND	ND	2665603562	166415833	1827321779	72213907	2521414970	82613478	1815592247	321236807
34	Hydrocarbon	Lepidozene	37.35	-	535073936	9674878	ND	ND	ND	ND	ND	ND	ND	ND	ND	ND
35	Hydrocarbon	Caryophyllene	37.74	1419	ND	ND	4125013071	1128174026	2199745596	171061482	2228232601	116801206	2593315957	84440692	2502931794	313207123
36	Hydrocarbon	Cedrene	37.85	1422	679834324	12270872	ND	ND	ND	ND	ND	ND	ND	ND	ND	ND
37	Hydrocarbon	Amopha-4,11-diene	37.98	-	161868612	37821311	ND	ND	ND	ND	ND	ND	ND	ND	ND	ND
38	Hydrocarbon	Alloaromadendrene	38.07	1461	ND	ND	ND	ND	2249335325	134173985	1216600162	112050097	2287446419	74207491	2194026024	36169616
39	Hydrocarbon	α-Funebrene	38.92	1389	2968143703	226213725	13997537725	641643711	16618962971	1856093170	37115636504	4253549591	20612097304	1634171085	37844330284	5902168701
40	Hydrocarbon	(+)-α-Himachalene	39.10	1542	1036735389	19036882	12139136871	303018726	4671549311	637362300	11841121350	312350898	10480767137	1086865438	12994792306	2475932045
51	Hydrocarbon	9-Octadecenenitrile	49.36	2084	77609075	2302748	ND	ND	ND	ND	ND	ND	ND	ND	ND	ND
6	Ketones	Sulcatone	25.74	-	3131183967	63972818	6101117594	234294975	5409393691	347220194	3626196850	100066620	4901664035	154186135	4412529284	180812701
11	Ketones	(+)-2-Bornanone	29.70	1144	164125048	2963656	1420710256	59822035	1564692131	98527825	1056027162	27716618	1210901115	39688454	1346891662	155376477
25	Ketones	2-Undecanone	33.58	1294	376304532	6858189	ND	ND	ND	ND	ND	ND	ND	ND	ND	ND
47	Ketones	Zingerone	41.45	1808	982348847	17839483	ND	ND	11628253685	1075691496	17246799521	740474778	ND	ND	ND	ND
53	Phenols	[6]-Gingerone	51.52	2396	99198537	21820415	837619799	183539079	715353469	57305849	721245530	21093770	1101371171	34025319	1138308208	158497502
54	Phenols	[6]-Shogaol	52.29	-	ND	ND	1222419821	144175430	1963427161	178004244	2707812328	71528361	1299210042	75309812	2866793909	844223762
